# Field test study on the evaluation of the microvibration controlling capacity of a mass concrete layer

**DOI:** 10.1038/s41598-022-23173-1

**Published:** 2022-11-24

**Authors:** Ming Zheng, Liangdong Zhuang, Jiansheng Fan, Yufei Liu, Jinlong Ren, Muning Rong, Wei Zhai

**Affiliations:** 1 Department of Civil Engineering, Tsinghua University, Beijing, 100084 China; 2 Beijing Construction Engineering Group Co., Ltd, Beijing, 100055 China

**Keywords:** Civil engineering, Environmental impact

## Abstract

Microvibration induced by natural disturbance and human activities has an adverse effect on the operation of the large-scale and ultraprecise facilities in the world. Under such circumstances, a passive vibration control method is generally deployed for such vibration-sensitive facilities, taking the High Energy Photo Source (HEPS) in Beijing as an example, a 3 m-thick mass concrete layer forming a ring foundation was cast at the facility, where a 1 m-thick reinforced concrete slab (RC slab) lies. Since microvibration control plays a crucial role in the operation of such large-scale scientific and ultraprecise facilities and few studies have been reported for large-scale concrete layer as antimicrovibration devices, this paper presents four field tests in Beijing, China, to evaluate the vibration control capacity of a mass concrete layer. Based on a large number of field tests, the effect of applying the concrete layer is discussed, and a reference is provided for the construction of similar facilities. The vibration signals, generated by shock excitation and ambient excitation, are measured through a highly sensitive and high-accuracy vibration acquisition system. It is concluded that the existence of the 1 m-thick RC slab has little influence on the microvibration signal frequency distribution in the vertical direction and that the signals from the concrete layer and subsoil differ by approximately 10 Hz in the vertical direction while differing by approximately 5 Hz in the horizontal direction. The microvibration control ability of the concrete layer is favorable in a higher frequency band over 20 ~ 30 Hz and more than 50% attenuation can be gained through the concrete layer; however, the microvibration control ability is not significant below 20 ~ 30 Hz. The vibration levels across different heights of the concrete layer section are the same. To prevent adverse vibration disturbance below 20 ~ 30 Hz, it is suggested that the traffic and road surface conditions should be taken into consideration when choosing the construction location. In addition, a long-term monitoring shows that 75% vibration energy at the site is firmly related to the construction activities which are approximately 1.4 km from the site.

## Introduction

As the exploration of the microworld is playing an increasingly important role in advancing human technology^1^, the construction of large-scale and high-precision scientific facilities is in a large demand. Under such circumstances, the synchrotron radiation facilities that have now advanced to the 4th-generation serve scientists’ requirements well. Such facilities take advantage of a phenomenon called synchrotron radiation, which is primarily observed through the electron or charged particles making a curvilinear motion that is accelerated close to the speed of light, and in this procedure, the electron or charged particles would emit electromagnetic radiation similar to a high energy X-ray machine^2^. To date, there are more than 60 such facilities in operation all around the world^3^.

Nevertheless, such facilities with high-precision are always vibration sensitive^2,7,8,10,11^ and generally, the higher the precision is, the stricter the vibration level. For the most advanced synchrotron radiation facilities, the maximum permissible microvibration level for displacement is usually confined to the nanometer scale, which often leads to a high cost to be spent on the microvibration control. In general, active or passive methods are two popular means used for vibration control. Although the active method is more effective than the passive method for low-frequency vibration control, it’s a more practical and economical to adopt a passive method for vibration control in the construction of large-scale and high-accuracy facilities^4–6^. To satisfy the stringent requirement for vibration control, different synchrotron facilities in the world have taken different methods during design and construction, which mainly focus on improving the property of the subsoil, including an engineered sand layer deployed in NSLS-II^7^, America; a combination of a 1.8 m concrete test plate and a gravel layer used in ALBA, Spain^8^; a 2 m-thick layer of soil–cement formed in Sirius, Brazil^9^; and piles and a thick test slab deployed in Diamond, Britain^10^.

**Figure 1:**
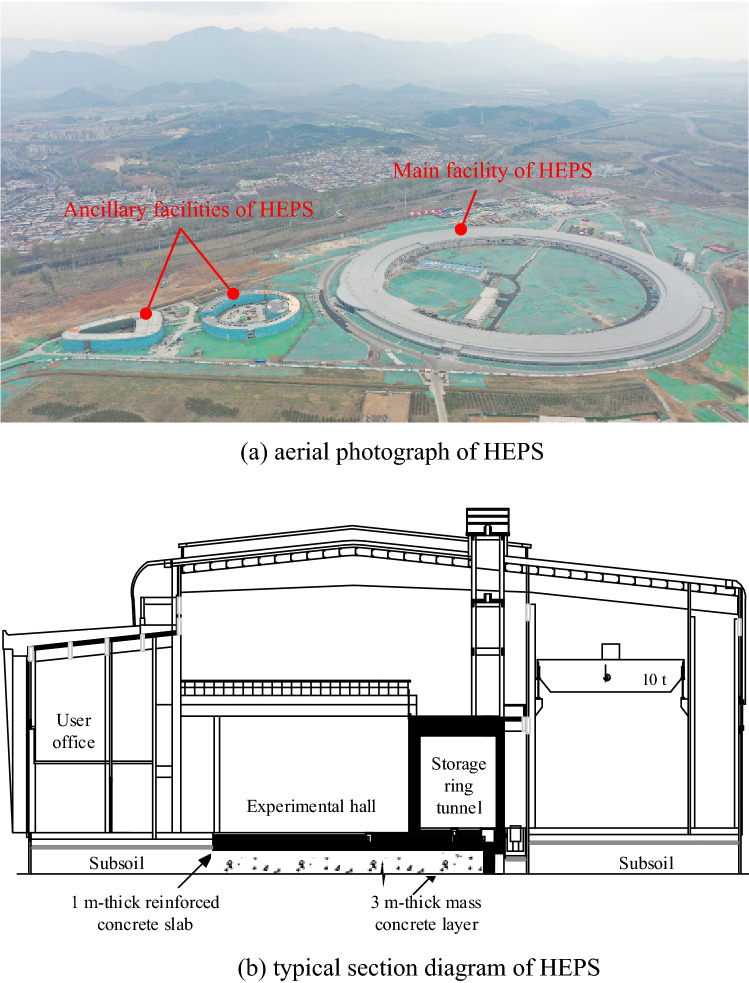
High Energy Photon Source in Beijing (under construction).

The High Energy Photon Source (HEPS, shown in Fig. 1) in Beijing is one of the most advanced 4th-generation synchrotron radiation facilities in the world, with an area of more than 125,000 m^2^. The vibration level of the HEPS’s experimental hall in Beijing is expected to be that the root-mean-square (RMS) value of the 1 s-sampling displacement in the frequency band of 1 ~ 100 Hz will be less than 25 nm after the construction is finished, which is stricter than the microvibration level measured at other sites^25^. To meet such a strict requirement, employing a vibration isolation trench around the facility may not be practical since a deep-depth trench is needed. However, employing a thick concrete layer can provide both satisfactory microvibration control ability and good base stiffness without necessitating subsoil application, which reduces construction costs and shortens the construction period. In this way, a 3 m-thick concrete layer forms the ring foundation with a diameter of approximately 400 m and is cast under a 1 m-thick reinforced concrete slab (RC slab), which serves as the floor of the experimental hall (shown in Fig. 1).

Usually, such a facility is constructed remote from any significant vibration sources nearby and is thus subjected to a complex ambient microvibration excitation. Studies^11,12^ indicate that ambient microvibration is composed of two parts: one is composed of natural phenomena at low frequencies (less than 1 Hz) such as earthquakes, wind, tides, rivers, rain, variations in atmospheric pressure, and the interaction of ocean waves with the costa-line; these are usually remote vibration sources. The other source is composed of human activities at high frequencies (greater than 1 Hz), such as air conditioning systems, road traffic, machinery, and pedestrians, and these vibration source influences are often associated with the local conditions. In 1968, Toksoz and Lacoss demonstrated that microvibration is an assemblage of body waves (primary wave and secondary wave) and surface waves (love wave and Rayleigh wave)^13^. To further study the property of vibration induced by human activities, detailed research on field test and numerical simulations of vibrations induced by traffic (trucks, buses, trains), or human walking has been conducted^14–23,28–31^.

Previous studies^14–23,28–31^ focused on specified vibration sources such as human walking or nearby traffic, but for large-scale and high-precision facilities, further attention needs to be paid to control over the ambient excitation’s influence, which can be treated as a wide-band white noise vibration signal. In addition, since construction of more than 125,000 m^2^ of the structure has been completed, the influence of such large- scale construction on the structure’s microvibration is not clear. Considering that microvibration in a large-scale construction site is a complex assembly of various vibration sources, not all of the factors can be taken into account in the numerical simulation or theoretical analysis, and field testing is the most intuitive and accurate method to study the microvibration characteristics during facility construction. Under such circumstances, various field tests and analyses have been conducted to identify the microvibration characteristics in the concrete layer, and the microvibration control performance of the slab are discussed under wide-band ambient excitation and a shock excitation. Moreover, efforts have been made to study the influences of the structure’s large-scale and long-period construction on the structure’s microvibration level. In addition, the test results can also be a reference for large-scale and high-precision facility construction in the future.

## Engineering background and testing device

Since this is the first time for a 4th-generation synchrotron radiation facility to cast such a mass concrete layer to help control the microvibration, the best way to evaluate the microvibration control capacity of the concrete layer is conducting comparable field tests at the construction site. The purposes for the field tests can be divided into verification purposes and comparison purposes: (1) verification: microvibration level verification by calculating the RMS value of displacement; (2) comparison: analysis of the characteristics of the microvibration signals around the site at different locations, different heights of the concrete layer and in different construction periods, which concentrate both on the time domain and on the frequency domain. The primary excitation methods are shock excitation and ambient excitation.

To evaluate the microvibration level at measurement points, the RMS value of displacement is calculated in the frequency domain according to Eq. (1) ^24^:

1 $$d_{{{\text{RMS}}}} \left( {f_{1} ,f_{2} } \right) = \sqrt {\int_{{f_{1} }}^{{f_{2} }} {S_{u} \left( f \right)df} } = \sqrt {\int_{{f_{1} }}^{{f_{2} }} {\frac{{S_{v} \left( f \right)}}{{4\pi^{2} f^{2} }}df} } = \sqrt {\sum\limits_{{f_{i} = f_{1} }}^{{f_{2} }} {\frac{{S_{v} \left( {f_{i} } \right)}}{{4\pi^{2} f_{i}^{2} }}\Delta f} } ,$$

where *d*_RMS_(*f*_1_, *f*_2_) represents the root mean square value of displacement in the frequency band from *f*_1_ to *f*_2_; *f*_1_ and *f*_2_ are the lower limiting frequency and upper limiting frequency, respectively; *S*_*v*_(*f*) (or *S*_*v*_(*f*_*i*_)) and *S*_*u*_(*f*) (or *S*_*u*_(*f*_*i*_)) represent the power spectrum density value of the velocity and displacement at frequency *f* (or *f*_*i*_), respectively; and ∆*f* = *f*_*i*+*1*_*−f*_*i*_ = (*f*_*2*_*−f*_*1*_)/*n*.

In addition, to evaluate the intensity of the measured vibration signals in the time domain, the RMS value of velocity is calculated according to Eq. (2) ^27^:

2 $$v_{{{\text{RMS}}}} \left( T \right) = \sqrt {\frac{1}{T}\int_{0}^{T} {v^{2} \left( t \right)dt} } = \sqrt {\frac{1}{T}\sum\limits_{i = 1}^{n} {v^{2} \left( n \right)\Delta t} } ,$$

where *v*_RMS_(*T*) represents the root mean square value of velocity in time period *T*; *T* is the time period of velocity in the times domain; *n* represents the sampling number during the time period *T*; and ∆*t* = *t*_*i*+1_−*t*_*i*_ = *T/n*.

**Figure 2:**
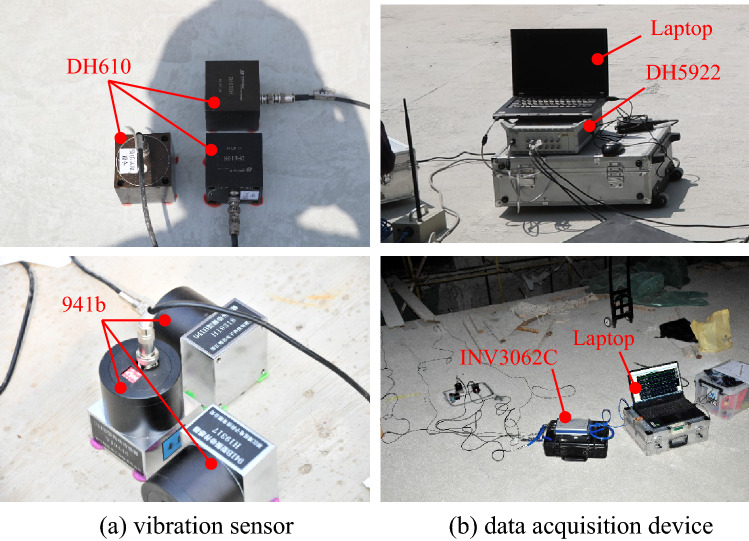
Vibrator sensor, data acquisition device.

During the tests, the sampling frequencies in tests I, II, III, and IV are 1000 Hz, 500 Hz, 256 Hz and 256 Hz respectively, which meet the requirements for the study of signals in both the frequency domain and the time domain. The test devices are mainly composed of data acquisition devices and vibration sensors, and details of the devices are listed in Table 1. The data acquisition device DH5922 and vibration sensor DH610 are products of Donghua Testing Technology Co., Ltd. The data acquisition devices INV3062C and INV3065N are products of the China Orient Institute of Noise & Vibration. Moreover, the vibration sensor 941b is a product of the Institute of Engineering Mechanics, China Earthquake Administration. To meet the requirements of the nanoscale displacement in the field test, the microvibration resolution capability of the devices is calculated according to the signal amplification factor and input noise of the data acquisition device: the input noise of INV3062C (shown in Fig. 2) is less than 0.6 μV, while the signal amplification factor is determined to be 100 (which means the range for the data acquisition device is selected as 100 mV), and the resolution for velocity is calculated according to Eq. (3)

3 $${\text{V}}_{{{\text{A/D - }}{\text{Re}} {\text{solution}}}} { = }\frac{{\text{U}}}{{2^{{\text{n}}} \cdot {\text{S}}}},$$

where *V*_A/D-Resolution_ represents the A/D resolution for velocity; *U* represents the measuring range of the data acquisition device; and *n* represents the AD digits of the data acquisition device. Moreover, supposing that the amplitude ratio between velocity and displacement is 2π*f*, the resolution for displacement in theory is less than 0.05 nm∙bit^−1^. Similarly, the input noise for displacement is calculated as follows: 0.24 nm for DH5922 with DH610, 0.04 nm for INV3062C with 941b, 0.14 nm for INV3065N with 941b, and the whole system satisfies the requirement for accurate measurement.

**Table 1:** Devices in field tests.

Device function	Model ID	Device sensitivity (V/m∙s^−1^)	Measurement range	A/D module	Pass band (Hz)	A/D resolution (nm∙s^−1^∙bit^−1^)	Input noise (μV_RMS_)	Field test application
Data acquisition device	DH5922	\	100 mV,1 V,10 V	24bits	\	0.30	≤ 3	I
	INV3062C	\			\	0.26	≤ 0.6	II, III
	INV3065N	\			\	0.26	≤ 1	IV
Vibration sensor	DH610	20	≤ 0.125 m s^−1^	\	1 ~ 100	\	\	I
	941b	23		\	1 ~ 100	\	\	II, III, IV

## Design and arrangement for field tests

**Figure 3:**
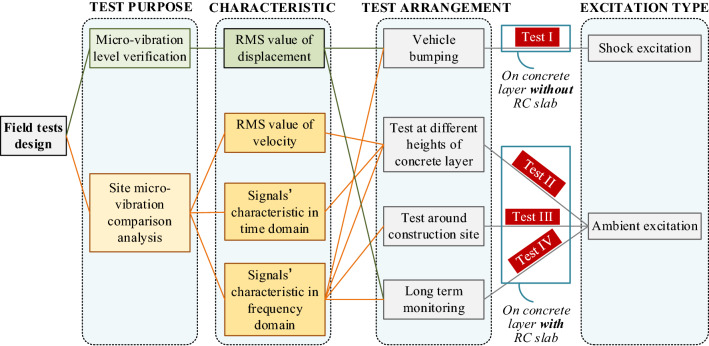
Field test arrangement.

**Figure 4:**
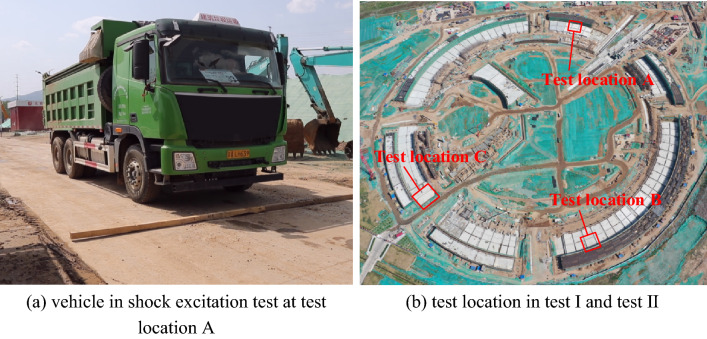
Vehicle in shock excitation test and test location.

**Figure 5:**
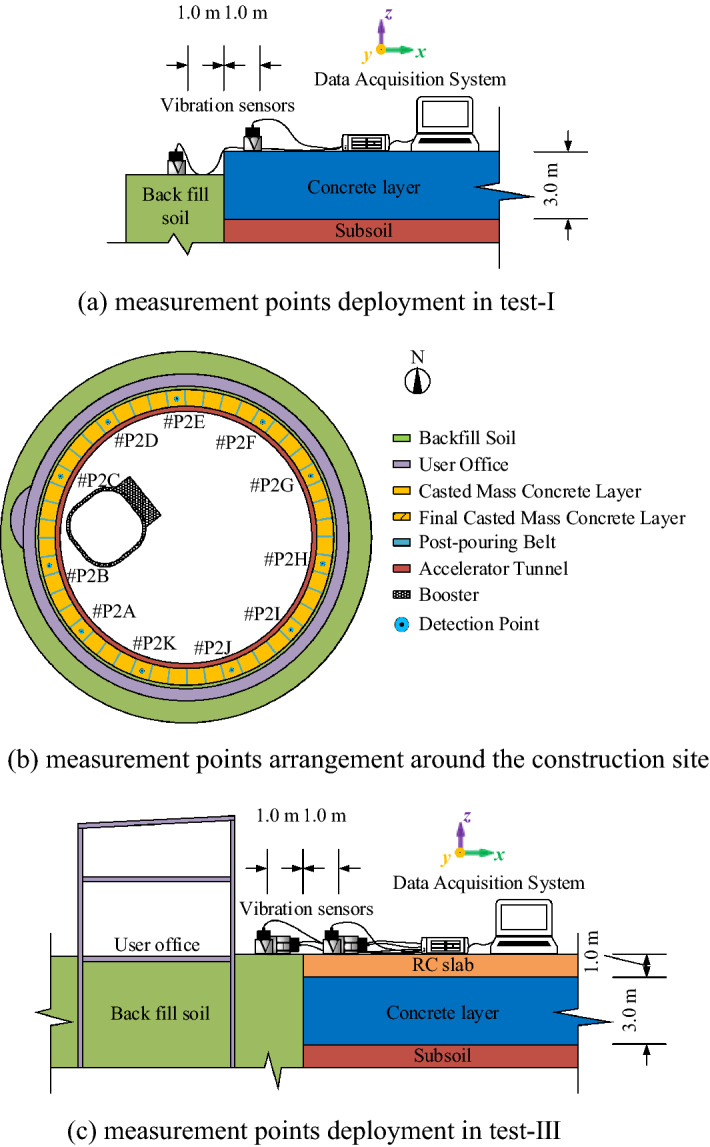
Measurement point arrangement.

A series of field tests were carried out, and the arrangement is shown in Fig. 3. Considering that the velocity provides the most compact presentation^26,27^, a velocity signal is acquired to assess the microvibration control capacity of the concrete layer. As shown in Fig. 3 and Fig. 4, in test I, during which the mass concrete layer was cast without an RC slab, to study the influence of the adjacent shock excitation on the concrete layer, a shock excitation test with a 10 t vehicle running over a wooden beam in test location A is carried out, and vertical vibration signals were measured on the surfaces of the concrete layer and adjacent soil to observe the vibration attenuation from the soil to the concrete layer under a sudden shock impact nearby. Subsequently, a measurement on the concrete layer under environmental excitation in test location B (shown in Fig. 4) is also conducted. In test II, when the RC slab is cast except at location C (shown in Fig. 4), which is a final cast part, to study the vibration signals’ characteristics at different heights of the concrete layer while considering that the signal differs at noon and night, the measurement at different heights of the mass concrete layer is conducted at test location C, and the test is performed separately at noon and at night. In test III, to evaluate the vibration signals’ characteristics between different construction periods of the whole concrete layer, two measurements under environmental excitation around the site (shown in Fig. 5) are completed after the RC slab is cast, and the measurement point includes the point at test location B. In this test, vibration signals in three directions are measured. The final concrete layer is cast after the 1st measurement around the site. In test IV, to evaluate the vibration level variation trend of the site over a long time, a long-term monitoring point is established that measures the vibration signals in three directions both on the RC slab and on the adjacent soil. In addition, signals of 24 h monitoring are also acquired at this point before the Spring Festival, when construction noise has the least impact.

**Figure 6:**
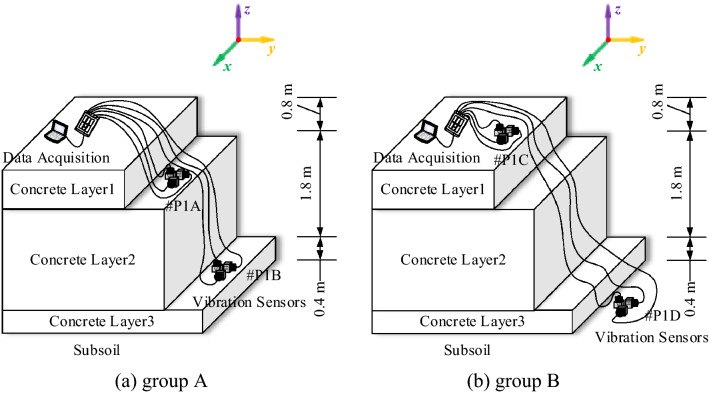
Detection point deployment in test location C.

**Figure 7:**
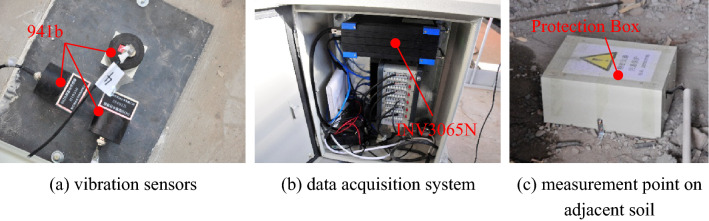
Long-term monitoring point arrangement at point #P2D.

The detailed test arrangement in test locations A and B for field test I and field test III is shown in Fig. 5. Two measurement points are deployed on the surfaces of the concrete layer and adjacent soil, while the vibration signals vertical to the ground are acquired. The test arrangement at test location C for field test II is shown in Fig. 6. To avoid noise from the construction activities, the test is conducted from 11:00 am to 13:00 pm and 10:00 pm to 12:00 pm. Moreover, restricted by the channel number of the data acquisition device, the measurement points are divided into 2 groups to study the influence of the thickness of the concrete layer on its microvibration control ability under ambient excitation. Each group contains two detection points, as #P1C and #P1D in group B are set on the concrete layer and subsoil surface, respectively, and the other two points in group A are set at different heights of the concrete layer. Since the mass concrete layer is cast in three layers from bottom to top, the vertical distance between adjacent measurement points is 0.8 m, 1.8 m, 0.4 m. Finally, a long-term monitoring point is set at the same position as #P2D to monitor the long-term vibration level at #P2D (shown in Fig. 7), and vibration signals in three directions from 11:00 pm to 5:00 am are acquired over 6 months. Monitoring is also conducted 24 h before the Spring Festival to determine whether the vibration level satisfies the design requirement.

## Test results and analysis

The test process, data and analysis are reported in detail in this section. Based on the excitation method and test location, this section is divided into four parts, while data and analysis are addressed both in the time domain and frequency domain. It should be noted that since the signals in 1 ~ 100 Hz are of concern, a bandpass digital filter of 1 ~ 100 Hz is applied to the obtained vibration signals. In addition, a noise signal of 50 Hz is obtained during the long-term monitoring, and a band-stop filter of 49 ~ 51 Hz is applied to the obtained signals.

### Test I: jumping tests of a motor vehicle

**Figure 8:**
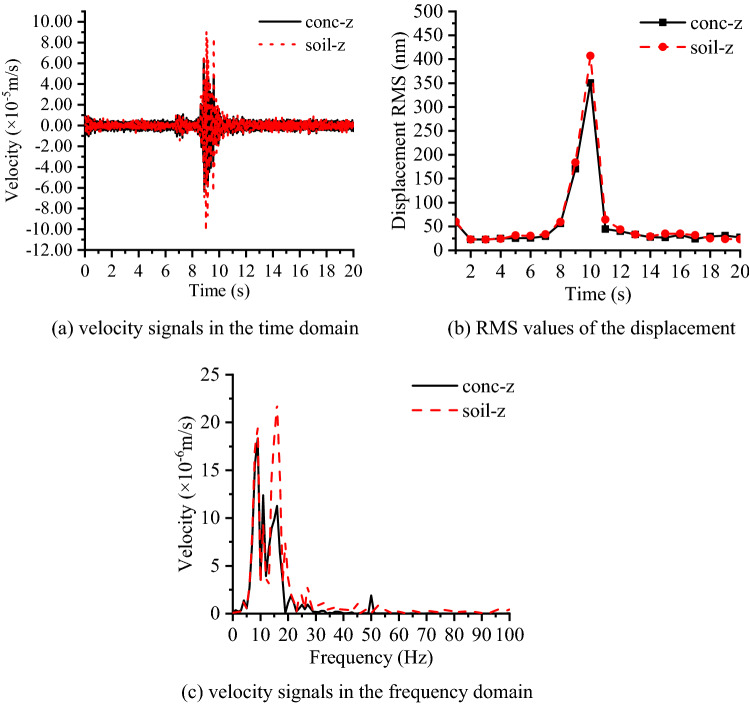
Signals in the time domain and frequency domain.

In test location A (shown in Fig. 4), a truck with a weight of 10 t running over a wooden beam mainly generates a sudden shock impact signal in the direction vertical to the ground, and the road is about 35 m to the concrete layer. The loading test is repeated for 12 tests, and the analysis selects the test that generates the most significant vibration signal. The symbol “conc-z” represents the vibration signal acquired on the concrete layer in the vertical direction, while “soil-z” represents the vibration signal acquired on the adjacent soil in the vertical direction. Figure 8(a) shows the time history curves of the vibration signal both on the soil and concrete layers, while the RMS value of displacement is also compared in Fig. 8(b). It can be found that the vibration response is more evident on the soil layer than that on the concrete layer when the vehicle runs over the wooden beam.

As shown in Fig. 8(c), to analyze the differences between the vibration signals measured on the concrete layer and on the adjacent soil when the vehicle runs over the wooden beam, the signals are converted into the frequency domain by the fast Fourier transform algorithm. Figure 8(c) shows that the vibration signal is distributed from 5 ~ 27.5 Hz in the soil layer and from 5 ~ 20 Hz in the concrete layer. Moreover, the measured vibration signals vertical to the ground differ in frequency band over 10 Hz, as the vibration signal on the concrete layer is larger than that on the soil surface in 10 ~ 13 Hz while being smaller over 13 Hz. In general, the RMS value of displacement decreases by 14% from the soil to the concrete layer. This phenomenon indicates that the shock excitation in the jumping tests of a motor vehicle may cause the vibration to be amplified in some frequency bands, which is related to the resonance frequency of the concrete layer at 10 Hz, while the RMS value of displacement is still decreased from the adjacent soil to the concrete layer. Since the microvibration level on the concrete layer obviously exceeds the microvibration control requirement, a similar shock impact should be avoided by restricting the deployment of the speed bump and limiting the irregularity of the road surface around the facility as well as the weight of vehicles that run near the facility.

### Test II: measurement at different heights of the concrete layer

As discussed in Section “Design and arrangement for field tests”, test II is divided into group A and group B. Group A is designed to study the vibration signals measured at different heights of the concrete layer, while group B is designed to evaluate the microvibration control ability of the concrete layer. Because the two groups in test II are not measured at the same time, the test conditions are different, and comparison between the two groups is not carried out.

#### Signal characteristic comparison in the time domain

The time histories of velocity measured at different test times, measurement points and directions in group A and group B are shown in Fig. 8 respectively. Table 2 lists the RMS values, variation ranges and average values of the velocity amplitude. Since the vibration source in this test is from the environmental excitation, it can be regarded as a stable random process that is approximately subject to the normal distribution. Because the mean value of the velocity is close enough to 0, which means it follows a normal distribution with a mean of 0, the mean value of velocity is not suitable to be chosen as a parameter to assess the microvibration control ability of the concrete layer. On this occasion, time history curves, RMS and variation of velocity values are combined for the assessment^26,27^. A large difference between the data acquired at noon and at night: the RMS values, variation values and average values of velocity signals measured at night are smaller than those measured at noon, and the noon-measured peak values of velocity are approximately 1 × 10^−5^ m/s, higher than those measured at night, which measure approximately 5 × 10^−6^ m/s. This proves that the human activities have an important influence on the vibration level, since there are no factories around the construction site and traffic and human activities nearby are mainly focused during the day time.

**Figure 9:**
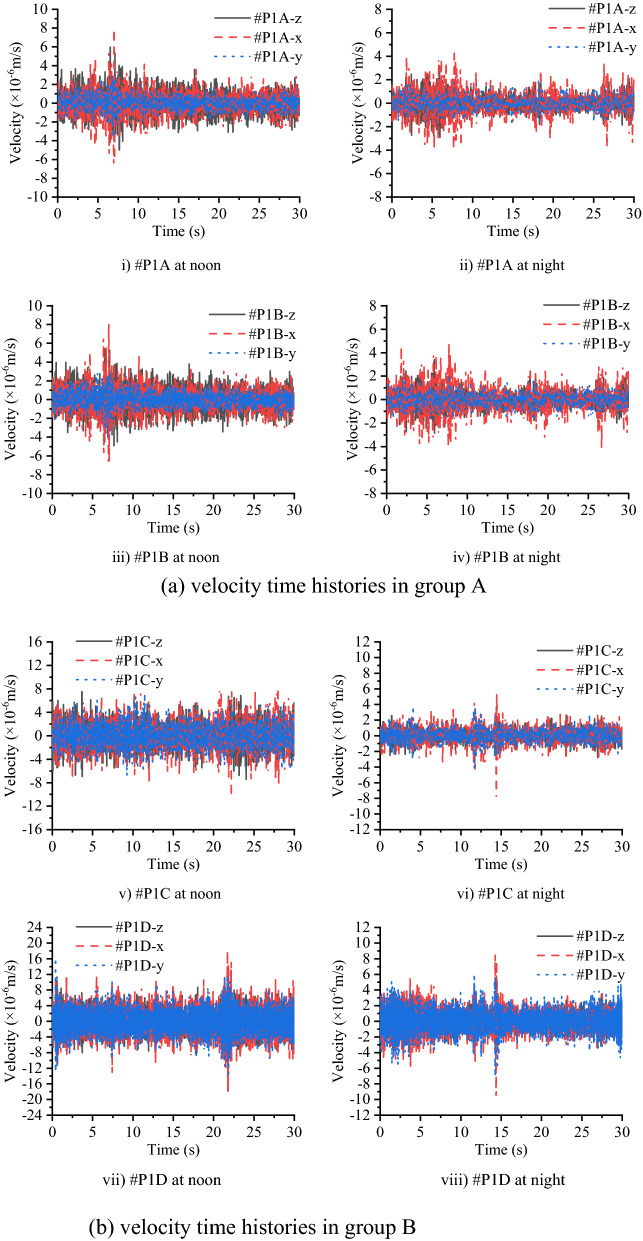
Velocity time histories in group A and group B.

**Table 2:** Root-mean-square value, variation range and average value of the measured velocity signals (m/s).

Test time	Test group	Measurement points	Variables	Direction		
				z	x	y
noon	group A	#P1A	RMS	1.138 × 10^−06^	1.239 × 10^−06^	6.839 × 10^−07^
			Variation range	1.031 × 10^−05^	1.444 × 1^−05^	6.532 × 10^−06^
			AVERAGE	3.336 × 10^−08^	− 1.343 × 10^−08^	1.450 × 10^−08^
		#P1B	RMS	1.189 × 10^−06^	1.293 × 10^−06^	6.883 × 10^−07^
			Variation range	1.031 × 10^−05^	1.490 × 10^−05^	6.810 × 10^−06^
			AVERAGE	1.618 × 10^−08^	− 1.529 × 10^−08^	4.052 × 10^−08^
	group B	#P1C	RMS	2.052 × 10^−06^	2.472 × 10^−06^	1.806 × 10^−06^
			Variation range	1.597 × 10^−05^	1.840 × 10^−05^	1.406 × 10^−05^
			AVERAGE	− 9.236 × 10^−09^	4.730 × 10^−08^	− 1.442 × 10^−07^
		#P1D	RMS	2.478 × 10^−06^	3.075 × 10^−06^	2.625 × 10^−06^
			Variation range	1.966 × 10^−05^	3.715 × 10^−05^	2.982 × 10^−05^
			AVERAGE	− 8.911 × 10^−09^	5.577 × 10^−10^	5.156 × 10^−08^
night	group A	#P1A	RMS	6.655 × 10^−07^	1.067 × 10^−06^	5.356 × 10^−07^
			Variation range	6.088 × 10^−06^	8.186 × 10^−06^	3.502 × 10^−06^
			AVERAGE	1.623 × 10^−21^	4.635 × 10^−11^	8.290 × 10^−11^
		#P1B	RMS	6.721 × 10^−07^	1.123 × 10^−06^	5.387 × 10^−07^
			Variation range	6.259 × 10^−06^	8.797 × 10^−06^	3.668 × 10^−06^
			AVERAGE	− 6.045 × 10^−12^	2.431 × 10^−10^	− 2.075 × 10^−11^
	group B	#P1C	RMS	7.441 × 10^−07^	1.159 × 10^−06^	7.930 × 10^−07^
			Variation range	6.513 × 10^−06^	1.337 × 10^−05^	7.771 × 10^−06^
			AVERAGE	3.575 × 10^−11^	2.859 × 10^−11^	1.183 × 10^−10^
		#P1D	RMS	8.842 × 10^−07^	1.406 × 10^−06^	1.298 × 10^−06^
			Variation range	7.616 × 10^−06^	1.820 × 10^−05^	1.314 × 10^−05^
			AVERAGE	− 8.620 × 10^−11^	7.319 × 10^−11^	4.304 × 10^−8^

In Fig. 9 in conjunction with Table 2, another notable phenomenon of the measured velocity on the concrete layer is that the signals in the y direction are smaller than those in the other two directions, which indicates that the concrete layer has a better vibration control ability in the y direction. This is related to the y direction being the hoop direction of the concrete layer; the length of the concrete layer in this direction is far longer than that in the other two directions, which increases the section stiffness in this direction, and enables the concrete layer to provide better performance in vibration control. In addition, the RMS values and variation ranges of the measured signals in the z direction on the subsoil are smaller than those in the x direction, which indicates that the vibration in the horizontal direction is worthy of attention in future studies.

**Figure 10:**
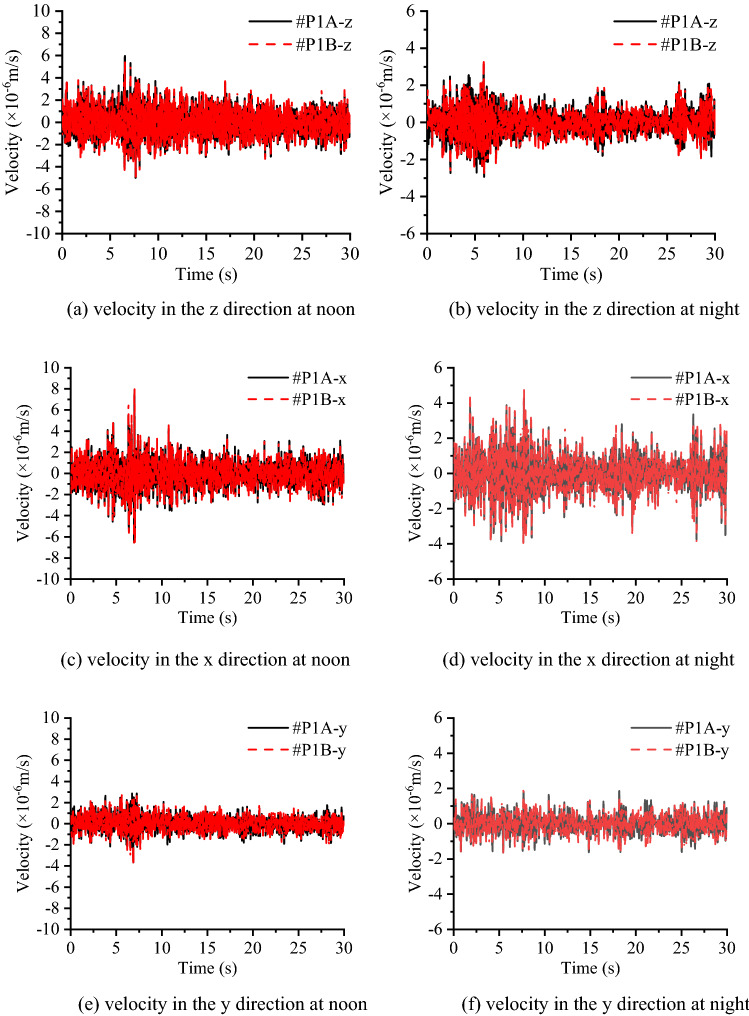
Comparison of the measured velocities at heights of 0.4 m and 2.2 m on the concrete layer in group A.

**Figure 11:**
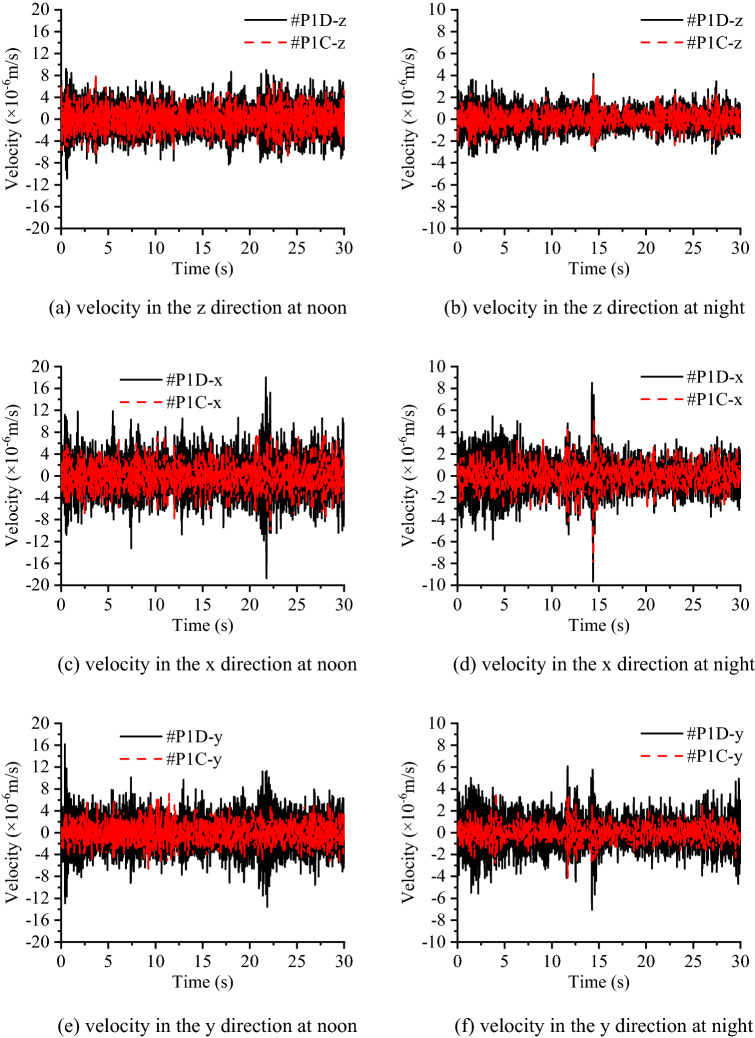
Comparison of measured velocities on the concrete layer and subsoil in group B.

Figure 10 compares the vibration signals measured at different heights of the concrete layer in the time domain, as #P1A is set at a height of 2.2 m and #P1B is set at a height of 0.4 m. Figure 11 compares the vibration signals on the top of the concrete layer and the subsoil in the time domain as well, while #P1C is set on the surface of the concrete and #P1D is set on the subsoil. Figure 10 shows that in each direction, the measured velocity signals tend to be the same, regardless of the test time. Since the RMS and variation of velocity in Table 2 are nearly the same and both time history curves at #P1A and #P1B can be seen clearly, these indicate that the cross section of the concrete layer can be regarded as a rigid body face with an identical rigid body movement at different heights. From Fig. 11 and Table 2, it can be found that the vibration level on the concrete layer is better than that on the subsoil since the RMS and variation of velocity values of #P1C are smaller than those of #P1D, while the time history curves of subsoil nearly cover all the curves of the concrete layer. This shows that the concrete layer is able to reduce the environmental vibration in the three directions.

#### Signal characteristic comparison in the frequency domain

**Figure 12:**
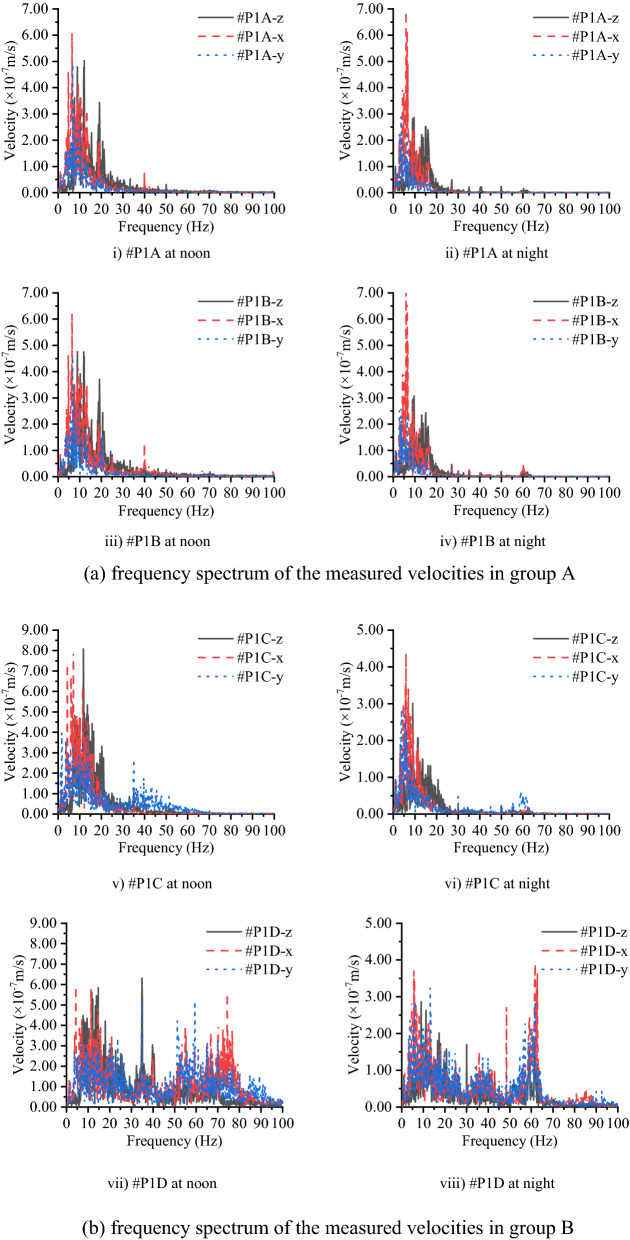
Frequency spectrum of the measured velocities on the concrete layer and subsoil in group A and group B.

To further understand the characteristics of the measured vibration signals, the velocity in the time-domain is converted to the frequency domain for analysis through the fast Fourier transform algorithm. Figure 12 shows the Fourier spectrum of the four measurement points at different testing times. The subgraphs i) to vi) show the difference of the signal measured in the three directions, which are measured on the concrete layer: the velocity signal in the y direction is smaller than those in the other two directions in the frequency band of 1 ~ 30 Hz, and in the frequency band of 30 ~ 100 Hz, the attenuation can be more than 50%. In addition, the signal in the y direction is approximately 70% of those in the z and x directions. Subgraph vii) and subgraph viii) in Fig. 12 show the vibration level for the velocity measured on the subsoil, and it can be found that the primary vibration signals are distributed in the frequency band below 95 Hz. Moreover, one noticeable phenomenon is that the vibration signal distribution range on subsoil in the z direction (vertical to the ground) is smaller (no more than 70 Hz) than those in the other two directions (below 85 Hz in x direction, below 95 Hz in the y direction). As previously mentioned, the vibration signal measured at noon is larger than those measured at night. Subgraphs vii) and viii) in Fig. 12 account for this phenomenon: vibration signals in 20 ~ 75 Hz decrease significantly at night and result in the vibration level at night being better than at noon, and this decreased frequency band relates to the vibration from traffic nearby. This shows that the high-frequency vibration decrease rapidly in the concrete layer, and the length of the concrete layer in the y direction provides a larger “stiffness” than in the other two directions, which leads to that the vibration signal being smallest in the three directions.

**Figure 13:**
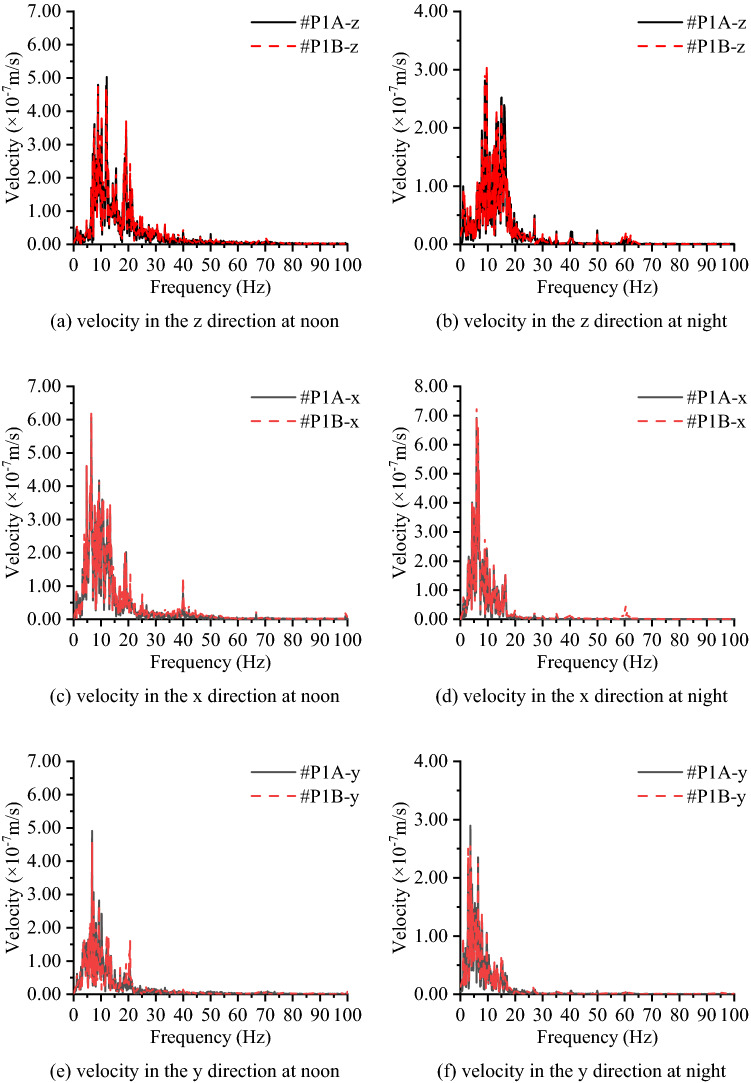
Frequency spectrum of the measured velocities on the concrete layer at a height of 0.4 m and 2.2 m on the concrete layer (group A).

**Figure 14:**
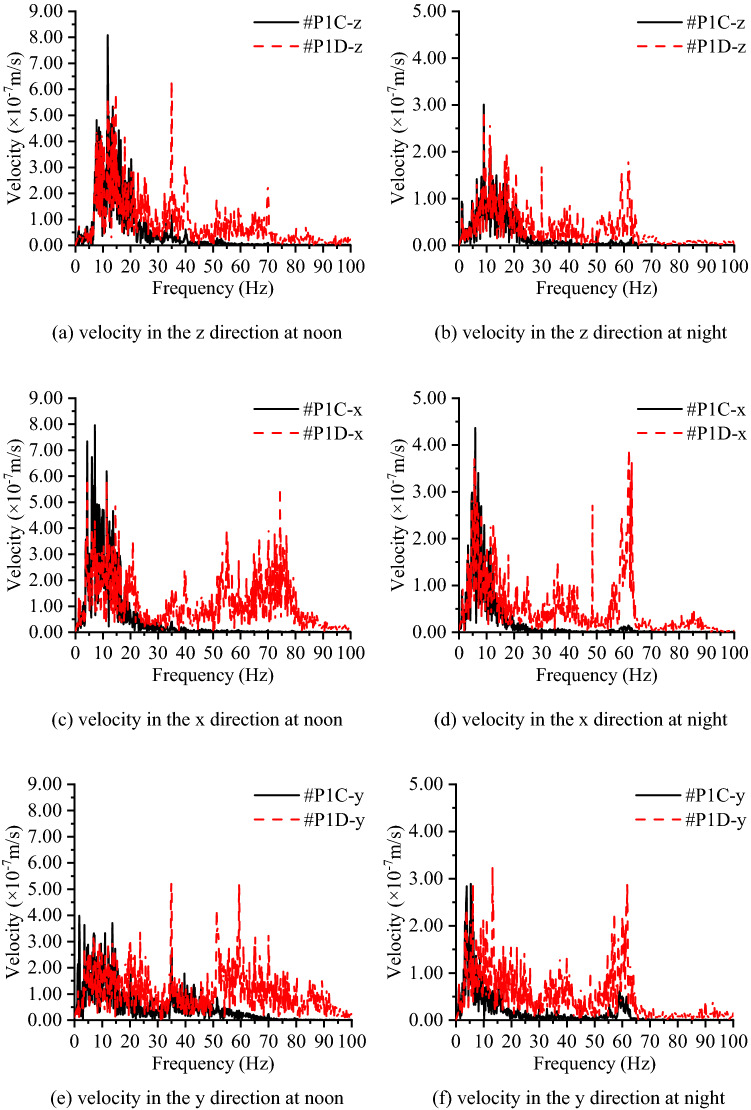
Frequency spectrum of the measured velocities on the concrete layer and subsoil (group B).

Figure 13 compares the vibration signals at different heights of the concrete layer in the frequency domain, where #P1A is set at a height of 2.2 m and #P1B is set at a height of 0.4 m, and Fig. 14 compares the vibration signal on the top of the concrete layer and the subsoil in the frequency domain, where #P1C is set on the surface of the concrete and #P1D is set on the subsoil. In the frequency domain, no significant critical separation can be found between the two curves, which means that the difference between the different heights of the concrete layer can be ignored, and the high-frequency components of the vibration are easily weakened by the concrete layer, while the low-frequency components are not.

### Test III: measurement at different locations on the concrete layer

**Figure 15:**
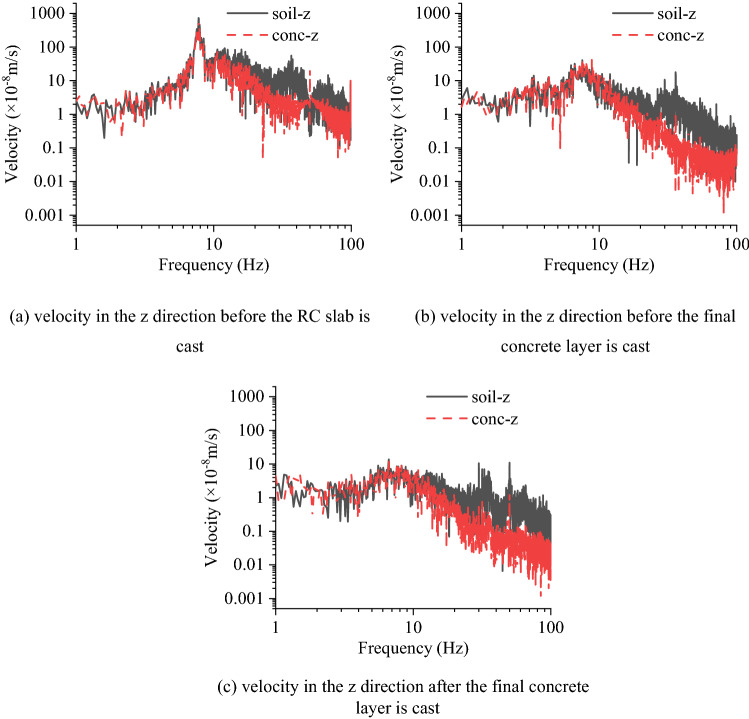
Frequency spectrum of the measured velocities on the concrete layer and adjacent subsoil in different construction periods at #P2I.

This test contains 2 measurements at different locations on the concrete layer and a measurement at test location B in test I during different construction periods. Since these measurements are acquired over a long time interval, a comparison between different measurements in the time domain is not carried out, as mentioned previously. Under such circumstances, the analysis in this test concentrates on the frequency domain. First, as the RC slab has not been cast, the measurement is conducted at test location B (shown in Fig. 4), which acquires the vibration signal in the x direction (corresponding to the radial direction) and z direction (corresponding to the vertical direction). Then, as the RC slab and the final concrete layer have been cast, two measurements at the same location and at other positions are conducted (shown in Fig. 5). To see the modification of the vibration during different construction periods, the vibration signals in the three directions are converted into the frequency domain in Fig. 15 with log–log graph axes to see changes of values for velocity as well as frequency. In Fig. 15, “z” corresponds to the vertical direction, “x” corresponds to the radial direction, and “y” corresponds to the tangential direction. The frequencies of the significant critical separation points of velocity signals are listed in Table 3.

**Table 3:** Critical frequency of the significant separation point between the signals (Hz).

Test time	Measurement points	2nd measurement direction			3rd measurement direction		
		z	x	y	z	x	y
Noon	#P2A	10	20	9	9	7	6
	#P2B	11	5	5	13	5	6
	#P2C	10	7	5	10	7	7
	#P2D	10	8	7	10	8	5
	#P2E	10	7	10	10	5	7
	#P2F	20	12	14	10	10	7
	#P2G	None	None	None	17	7	8
	#P2H	10	10	8	9	6	5
	#P2I	9	6	6	10	6	5
	#P2J	10	7	8	10	6	8
	#P2K	10	7	5	12	8	7
Night	#P2A	14	6	5	9	5	7
	#P2B	9	7	4	10	5	6
	#P2C	10	7	6	10	5	7
	#P2D	10	5	6	14	6	5
	#P2E	15	8	10	11	5	6
	#P2F	13	10	10	11	6	6
	#P2G	10	5	5	10	5	7
	#P2H	10	14	10	9	5	8
	#P2I	9	7	6	12	8	8
	#P2J	12	6	6	13	6	5
	#P2K	9	3	5	9	6	6

As shown in Fig. 15, the symbol “conc-z” represents the vibration signal measured on the concrete layer/RC slab in the vertical direction. After the RC slab is cast, the curves for vertical vibration signals measured on the concrete layer and soil differ mainly over 10 Hz and the curves show an evident separation of signals measured on the concrete layer to signals measured on the subsoil. This indicates that the vibration control capacity of the concrete layer performs well for vibrations in the frequency band over 10 Hz. In addition, after the final concrete layers have been cast, no significant shift of the vertical vibration signal in the frequency domain is observed, which means that the integrity of the concrete layer has no significant influence on the vertical vibration signals measured on the already cast concrete layer. Furthermore, as shown in Table 3, the frequencies of the significant critical separation point between signals measured on the RC slab and adjacent soil vary from 9 ~ 15 Hz in the z direction and 5 ~ 10 Hz in the x and y directions. This phenomenon is related to the vibration levels at different locations of the large-scale construction site being slightly different during the test time. In addition, the frequencies of the significant critical separation point in the horizontal direction are smaller than those in the vertical direction, which is associated with the large difference in the section sizes in different directions.

### Test IV: long term monitoring

**Figure 16:**
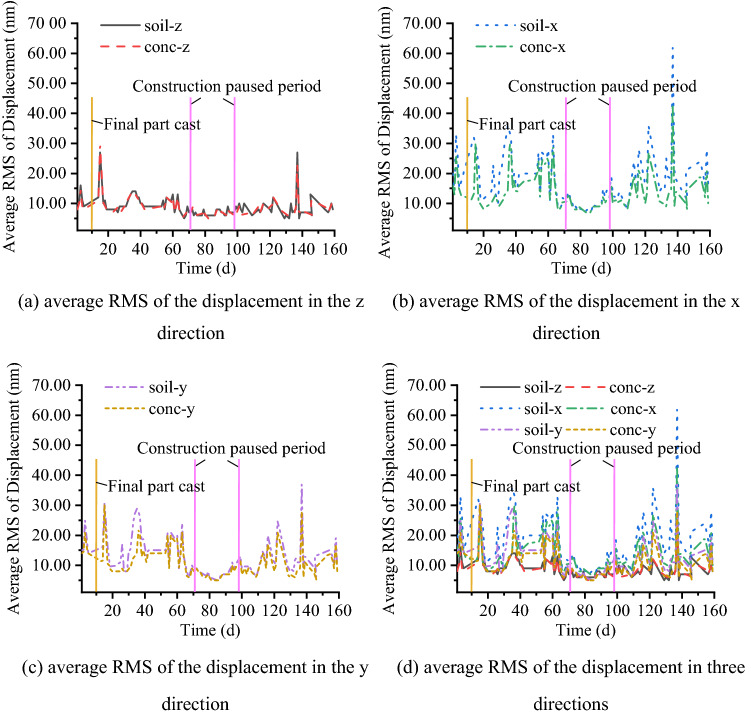
Average RMS value of the displacement at night.

**Figure 17:**
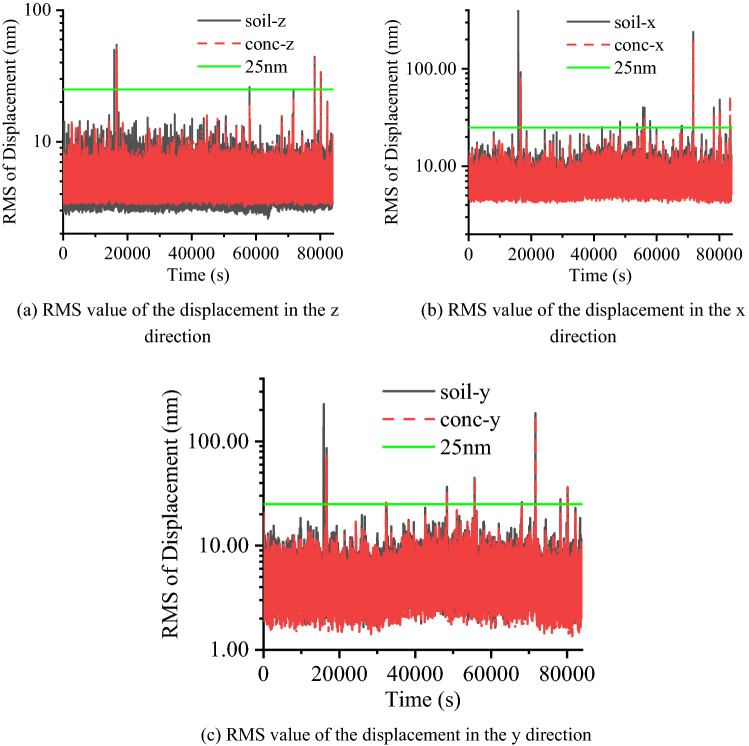
Displacement RMS value at each second over 24 h.

At the long-term monitoring point, two measurement points are deployed that measure vibration signals in three directions. As mentioned in the preceding section, the “z” direction corresponds to the vertical direction, the “x” direction corresponds to the radial direction, and the “y” direction corresponds to the tangential direction. The symbol “conc-z” represents the vibration signal measured on the RC slab in the vertical direction in Figs. 16, 17. The long-term monitoring lasts for 159 days, and starting at 11:00 pm and ending at 5:00 am the next day, during which time the construction activities all around the site are stopped. In addition, since the monitoring period contains a Spring Festival vacation, the monitoring activities last for 24 h during this period.

**Figure 18:**
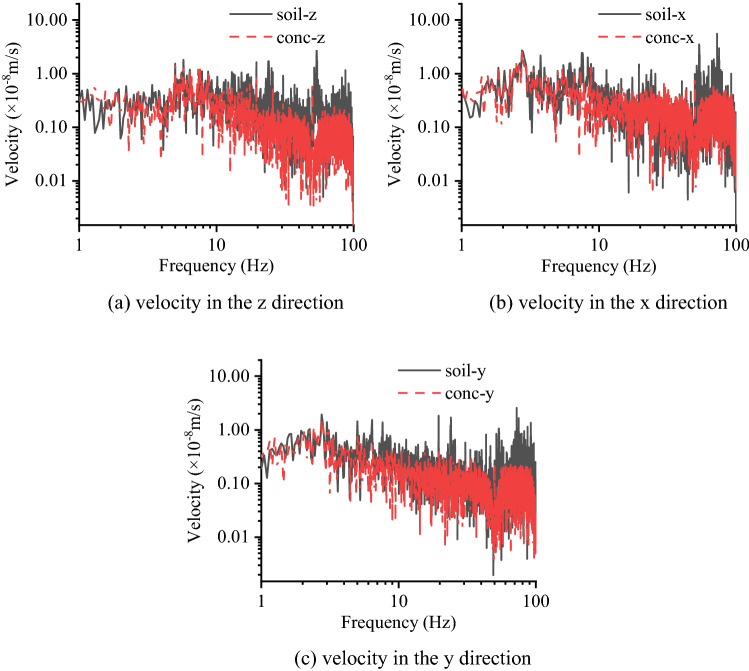
Frequency spectrum of the measured velocities at 4:00 at the long term monitoring point.

To observe the variation in each night’s vibration level during long-term motoring, the average displacement RMS values are calculated. As shown in Fig. 16, it can be found that the average RMS values of displacement in the horizontal direction are larger than those in the vertical direction. In addition, the average RMS values of displacement during day 68 and day 82 show a declining trend, which is related to the decrease in human activities and is especially evident horizontally. During day 76 and day 91, the average RMS values of displacement are approximately 25% of the curves’ peak value, which corresponds to a total pausing of the construction activities around the site near 1.4 km. In addition, during this time, the vibration level satisfies the microvibration control requirement. To further study the vibration level in a whole day when there are no human activities in the construction site, a 24 h monitoring is carried out before the traditional Chinese New Year’s Eve, which starts at 8:25 pm. As shown in Fig. 17, the RMS values of displacement in three directions are calculated, and most of the time the vibration level satisfies the microvibration control requirement, which is plotted as a green line. However, at some moment, there is a sudden change in the vibration level due to a vehicle passing by the monitoring point on the RC slab and generating a sudden shock impact. Moreover, it can be found that at the specific moment when a sudden shock impact occurs, the vibration signals measured in the horizontal direction are more evident than those measured in the vertical direction, which is worth addressing in the future studies. The 24 h-signals are converted into the frequency domain to compare the difference between the signals measured on the RC slab and on the soil. The results are listed in Table 4, where Fig. 18 shows the frequency spectrum of the signals at 4:00. It can also be found that during the day before the traditional Chinese New Year’s Eve, the vibration signals on the soil and RC slab differ by 9 ~ 10 Hz in the vertical direction while differing by 3 ~ 6 Hz in the horizontal direction.

**Table 4:** Critical frequency of the significant separation point between the signals (Hz).

Time	Measurement direction			Time	Measurement direction		
	z	x	y		z	x	y
8:00 pm	10	5	5	8:00 am	10	5	3
9:00 pm	10	6	3	9:00 am	10	5	4
10:00 pm	10	5	5	10:00 am	9	4	5
11:00 pm	9	6	4	11:00 am	9	6	4
12:00 pm	10	5	5	12:00 am	10	5	4
1:00 am	10	5	3	1:00 pm	10	5	3
2:00 am	9	5	3	2:00 pm	10	5	4
3:00 am	9	5	5	3:00 pm	10	5	5
4:00 am	9	5	5	4:00 pm	10	5	5
5:00 am	9	6	4	5:00 pm	10	5	5
6:00 am	9	5	5	6:00 pm	10	6	3
7:00 am	10	4	4	7:00 pm	10	4	3

## Conclusions

Four field tests are conducted in Beijing, China, to evaluate the microvibration control capacity of the concrete layer with an RC slab. The main conclusions are listed below:

- (1) The cast for a 1 m-thick RC slab has little influence on the microvibration signals measured in the vertical direction. However, the signals differ by approximately 10 Hz in the vertical direction while differing by approximately 5 Hz in the horizontal direction relative to the section size in the corresponding direction.
- (2) The concrete layer provides a better microvibration control capacity in a higher frequency band over 20 ~ 30 Hz, and the attenuation through the concrete layer can be more than 50%.
- (3) Under ambient excitation, the microvibration levels at different heights of the concrete layer are the same, which indicates that the whole section of the concrete layer can be regarded as a rigid face in the same direction as the concrete layer subjected to ambient excitation.
- (4) To obtain a better microvibration level, the building site should be far from roads with heavy traffic, which will generate adverse vibration disturbance below 20 ~ 30 Hz. Heavy traffic should also be prevented during the operation of the facility, which may lead to a shock excitation situation near the facility.
- (5) The microvibration level is affected by construction activities, which are 1.4 km from the site, and the microvibrations in the horizontal direction are worthy of attention. Approximately three-quarters of the vibration energy at the site shows a strong relation with the construction activities nearby.

## Data Availability

The datasets used and/or analyzed during the current study are available from the corresponding author on reasonable request.
